# Recurrent exercise-induced acute kidney injury by idiopathic renal hypouricemia with a novel mutation in the *SLC2A9* gene and literature review

**DOI:** 10.1186/1471-2431-14-73

**Published:** 2014-03-14

**Authors:** Huijun Shen, Chunyue Feng, Xia Jin, Jianhua Mao, Haidong Fu, Weizhong Gu, Ai’min Liu, Qiang Shu, Lizhong Du

**Affiliations:** 1 Department of Nephrology, The Children’s Hospital of Zhejiang University School of Medicine, Hangzhou 310003, Zhejiang Province, China; 2 Department of Pathology, The Children’s Hospital of Zhejiang University School of Medicine, Hangzhou 310003, China

**Keywords:** Idiopathic renal hypouricemia, Acute kidney injury, *SLC2A9*, Gene mutation

## Abstract

**Background:**

Idiopathic renal hypouricemia (iRHUC) is an autosomal recessive hereditary disorder, characterized by impaired tubular uric acid transport, re-absorption insufficiency and/or the acceleration of secretions. Some patients present with severe complications, such as exercise-induced acute kidney injury (EIAKI) and nephrolithiasis.

**Case presentation:**

Herein, we report the case of a girl with severe iRHUC (serum urate 0.05 mg/dL, fractional excretion of uric acid 295.99%) associated with recurrent EIAKI, in whom the disease was caused by a homozygous mutation (g.68G > A in exon 3) in the *SLC2A9* gene. Her family members (father, mother and brother) carried the same mutation but were heterozygous, without any signs of severe hypouricemia.

**Conclusions:**

Our findings indicate that iRHUC is a rare disorder but that it should also be considered in patients with EIAKI, especially in those patients who manifest with moderately elevated or normal serum concentrations of uric acid during the acute phase of AKI. Mutational screening of the *SLC2A9* gene is necessary for the diagnosis of iRHUC, and homozygous mutations of the *SLC2A9* alleles can cause severe hypouricemia. Careful attention should be paid to any signs of hypouricemia during the recovery phase of AKI and long-term follow-up.

## Background

Idiopathic renal hypouricemia (iRHUC) is an autosomal recessive hereditary disorder characterized by impaired tubular uric acid (UA) transport, reabsorption insufficiency and/or the acceleration of secretion [1-4]. Currently, there are two subtypes of RHUC. Type 1 (RHUC1) is characterized by loss-of function mutations in the *SLC22A12* gene, which encodes urate transporter 1 (URAT1). Mutations in the *SLC22A12* gene are responsible for most cases of renal hypouricemia. In contrast, type 2 (RHUC2) was recently revealed to be caused by defects in the *SLC2A9* gene [5]. Most of these patients are clinically asymptomatic and are detected incidentally, but some have nephrolithiasis or hematuria or are predisposed to exercise-induced acute kidney injury (EIAKI). Dinour et al. reported that homozygous mutations of *SLC2A9* cause more severe hypouricemia than URAT1 mutations and are associated with a high incidence of renal calculus and EIAKI [6].

The diagnosis of iRHUC is based on biochemical markers: hypouricemia (<119 μmol/L or 2.0 mg/dL) and increased fractional excretion of uric acid (FE-UA) (>10%) [7]. Furthermore, the exclusion of secondary causes of hyperuricosuric hypouricemia (such as Wilson’s disease, Fanconi syndrome and drug-induced tubulopathy) is very important. Confirmation of the diagnosis is accomplished by molecular analysis of the *SCL22A12* and/or *SLC2A9* genes.

To present time [8], only more than 100 patients with *SLC22A12* mutations, and a few patients with *SLC2A9* defects have been characterized worldwidely. Here, we describe one case of idiopathic renal hypouricemia; the patient presented with recurrent EIAKI and had a novel homozygous nonsense mutation g.68G > A in exon 3 of the *SLC2A9* gene.

## Case presentation

### First episode

An otherwise healthy 12.1-year-old girl presented to our clinical department on October 22, 2010, with nausea, vomiting and acute central abdominal pain for 4 days after an 800-meter run. Her parents denied any illness, viral prodrome or aminoglycoside or nonsteroidal anti-inflammatory drug ingestion prior to exercising. Her urea nitrogen is 32.62 mmol/L, creatinine 663.1 μmol/L (1 mg/dl = 88.4 μmol/L) and uric acid 201.8 μmol/L (1 mg = 59.48 μmol/L). Thus, she was admitted to our ward with acute kidney injury on October 25. Her past medical history was otherwise unremarkable. There was no family history of muscle disorders or renal diseases.

On physical examination, her blood pressure was 139/90 mmHg without orthostasis. Her weight and height were 40 kg and 146 cm, respectively. The examination was entirely unremarkable except for a mild lethargy. There was no muscle swelling or tenderness.

The laboratory evaluation revealed the following results: pH 7.443, potassium 4.7 mmol/L, bicarbonate 16.5 mmol/L, SBE -6.8 mmol/L, creatine kinase 43 U/L (normal range 30–200 U/L), serum myoglobin 25.1 μg/L (normal range 0.1-70 μg/L), and blood β2-microglobulin 4233 ng/mL. The urinalysis revealed a specific gravity of 1.020, a pH of 5.5-5.0, 0–4 red blood cells/μL, rare renal tubular epithelial cells and rare uric acid crystals. No glycosuria, aminoaciduria or crystallization was found. The 24-hour urinary protein and urine output were 604.3 mg and 1500 mL, respectively. Her urinary secretory IgA was 1.13 μg/mL (normal range 0.74-2.5 μg/mL), a_1_-microglobulin >32.8 mg/L (normal range 1.36-10.36 mg/L), microalbumin >50 μg/mL (normal range 3.03-16.81 μg/mL) and immunoglobulin G 30 μg/mL (normal range 2.29-5.45 μg/mL). The following parameters were within normal limits or negative: serum immunoglobulin; serum complement titer; antinuclear antibody (ANA); double-stranded DNA (dsDNA); myeloperoxidase-antineutrophil cytoplasmic antibody (MPO-ANCA); perinuclear-antineutrophil cytoplasmic antibody (P-ANCA); cytosolic-antineutrophil cytoplasmic antibody (C-ANCA); PR3-ANCA; anti-streptolysin O antibody (ASO); HIV; EBV; TORCH; anti-glomerular basement membrane antibodies; HBsAg; and hepatitis C antibody. Tests with pyrazinamide or probenecid were not performed.

A renal biopsy was performed on day 9 after admission. The light microscope revealed normal glomeruli and arterioles, with vacuolar degeneration in the tubular epithelial cells, accompanied by interstitial edema, scattered lymphocytes and monocyte infiltration. Immunofluorescence stainings for IgG, IgA, IgM, C3, C4 and Fib were all negative. Electron microscopy showed focal effacement of foot processes and a normal basement membrane without any electron-dense deposits. These results revealed the kidney to be recovering from acute tubular necrosis.

With conservative therapy of fluid control, her renal function recovered gradually. After 15 days of admission, she was discharged with improved renal function (BUN 10.52 mmol/L and serum creatinine 106.9 μmol/L). During the 1-year follow-up, her eGFR calculated by the Schwartz formula [9] eventually recovered.

### Second episode

Unfortunately, a relapse of EIAKI occurred nearly 1 year after the first episode. On December 23, 2011, the patient was admitted again to our hospital with nausea, vomiting, abdominal pain and loin pain after the same exercise. The physical examination did not reveal any abnormalities. Her blood pressure was 94/51 mmHg, and her 24-hr urinary output was 1400 mL and 24-hr urine protein excretion was 220 mg. In addition, Scr was 143 μmol/L, BUN was 12.59 mmol/L, and uric acid was 21 μmol/L. The serum copper ceruloplasmin was normal, and serum myoglobin was 21.3 μg/L. The 24-hour urinary excretion of calcium and copper was within the normal range. The renal ultrasound scan showed increased echogenicity in the cortex of both kidneys, but hydronephrosis was not observed. Her MRI was normal.

**Figure 1:**
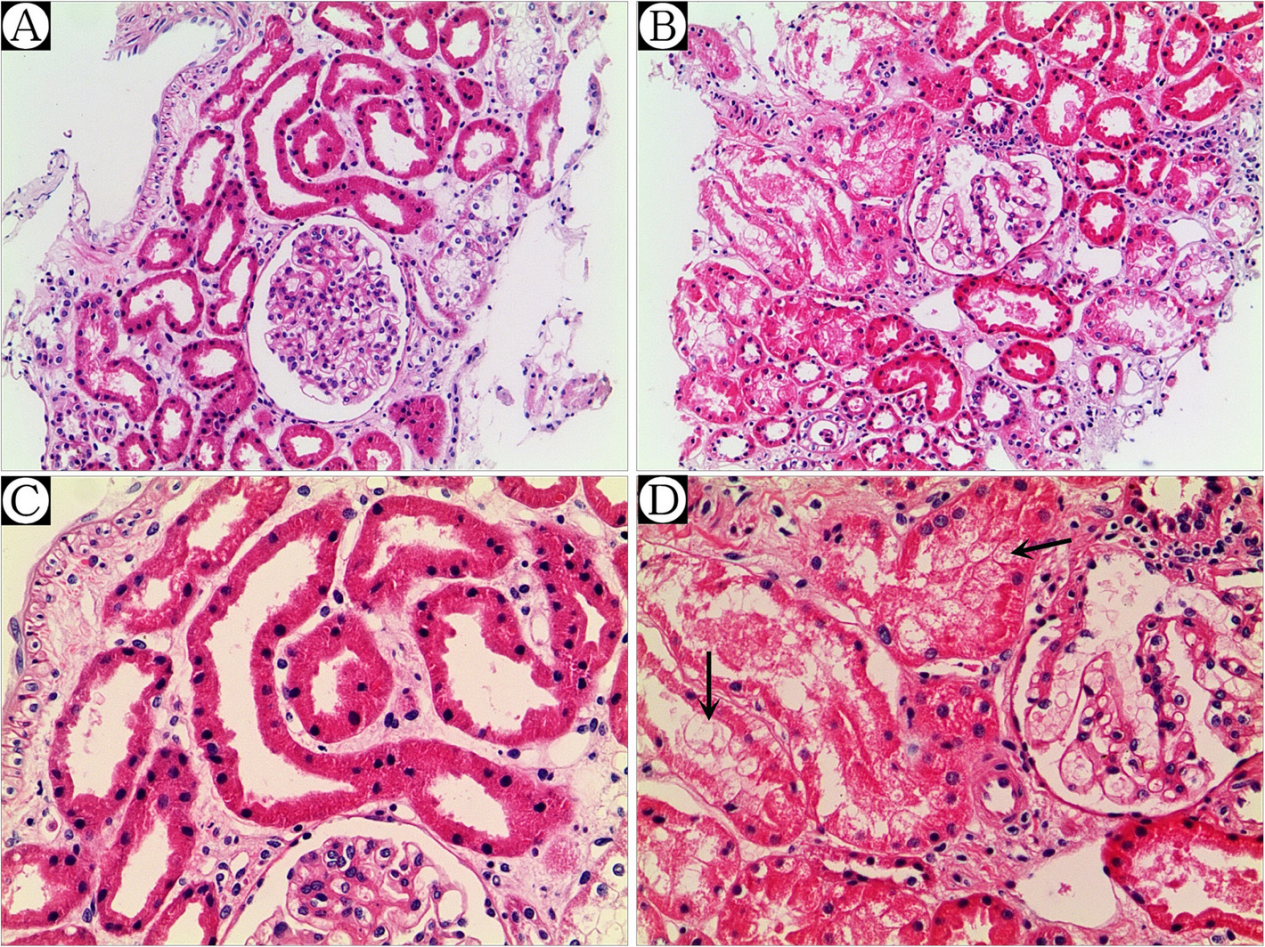
**Secondary renal biopsy revealed very slight mesangial cell proliferation and normal arterioles by light microscope, with moderate vacuolar degeneration in the tubular epithelial cells (↖), accompanied by interstitial edema and small amounts of lymphocytes and monocyte infiltration. A** &**B** × 100, and **C** &**D** × 200.

A second renal biopsy was conducted on December 28, 2011. Very slight mesangial cell proliferation and normal arterioles were observed by light microscope, with moderate vacuolar degeneration in the tubular epithelial cells, accompanied by interstitial edema and small amounts of lymphocytes and monocyte infiltration (Figure 1). Immunofluorescence stainings for IgG, IgA, IgM, C3, C4 and Fib were all negative. The present renal tubular-interstitial lesions appeared more moderate than previously.

**Figure 2:**
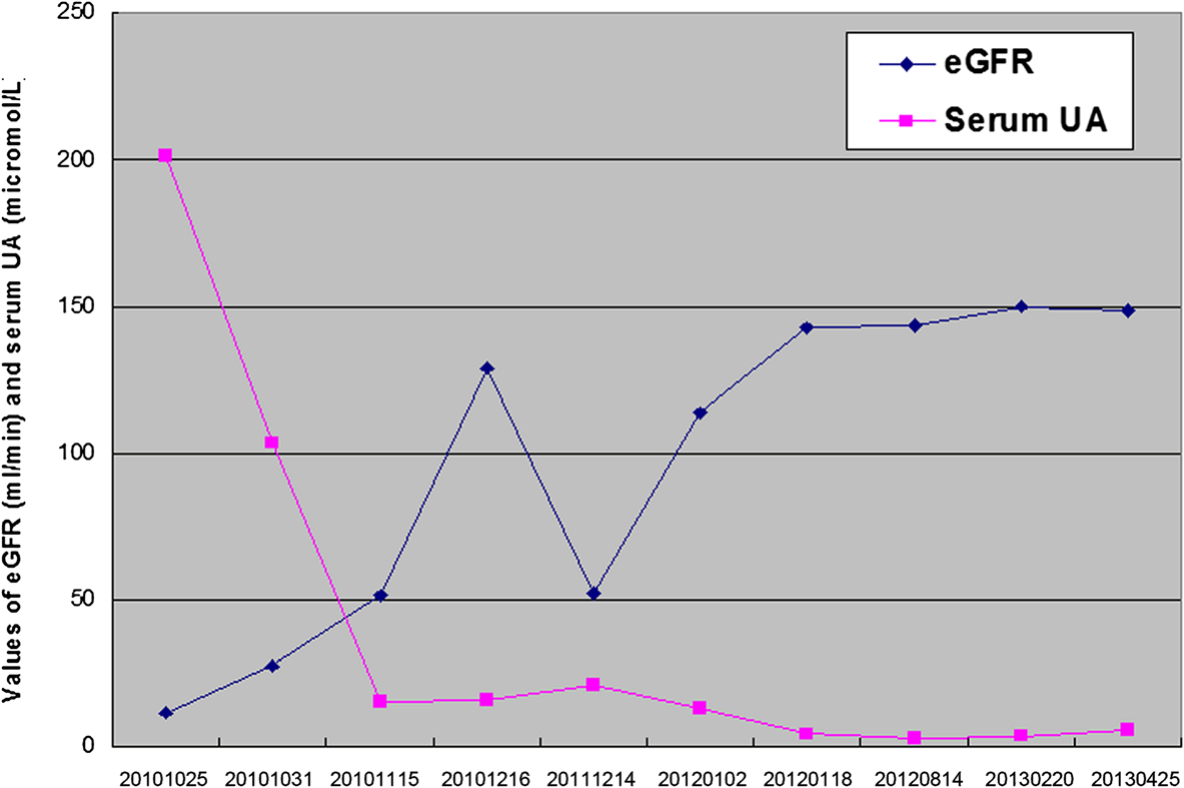
**Variation of eGFR (ml/min) and serum uric acid (UA, μmol/L) within 2.5-year follow-up in patient with idiopathic renal hypouricemia.**

This time, we noticed an extremely low concentration of uric acid in the serum. We performed a fractional excretion of urate (FEUA), and the result was 295.99%. All secondary causes of renal hypouricemia and EIAKI were excluded. A diagnosis of EIAKI by idiopathic renal hypouricemia was made retrospectively. After symptomatic treatment, the patient was discharged on day 13. During the follow-up, she was revealed to have normal Scr and eGFR, but severe hypouricemia (3.1–8.7 μmol/L) remained. Anaerobic activity was strictly prohibited, and no further episode of EIAKI occurred despite persistent hypouricemia at the 1.5-year follow-up. The laboratory findings of Scr and serum uric acid from each hospitalization are summarized in Figure 2.

The serum UA level was also screened in other family members. Her parents’ serum urate levels were 220.6 μmol/L (mother) and 263.4 μmol/L (father). Her younger brother’s (8 years old) serum urate level was 115.6 μmol/L, and his fractional excretion of urate was 24.72%.

### Mutational analysis

**Figure 3:**
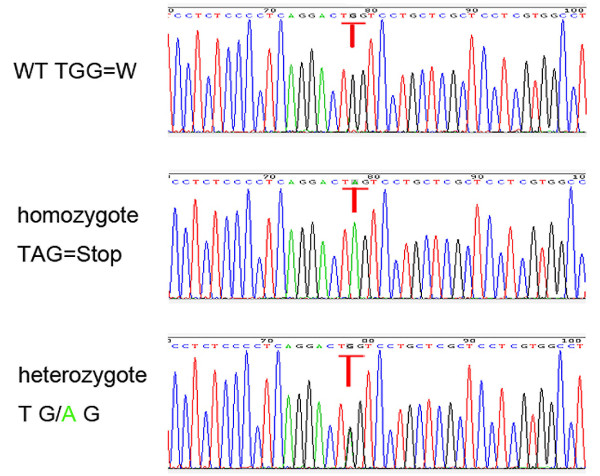
**Electropherograms of partial sequences of exon 3 of *SLC2A9 *** **showing a novel homozygous mutation g.68G > A (p.Trp23Stop) in patient exhibiting clinical features compatible with idiopathic renal hypouricemia and same but heterozygous mutation in her father, mother and brother.**

**Figure 4:**
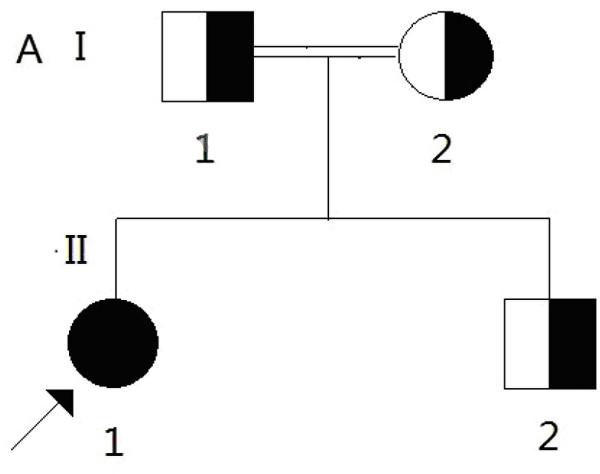
**Pedigree of the family carrying novel homozygous or heterozygous mutation in *SLC2A9 *gene.** The results of the analysis in the patient’s family members suggested an autosomal recessive mode of inheritance.

Secondary RHU, caused by isolated or generalized tubular defects, such as Fanconi syndrome, Wilson disease, cystinosis, heavy metal poisoning or drug-induced tubulopathy, was excluded before the molecular analysis of the *SLC22A12* and *SLC2A9* genes in the present study. Sequence analysis of the *SLC22A12* and *SLC2A9* genes was performed in the patient and her family members under the diagnosis of EIAKI associated with idiopathic renal hypouricemia. The clinical data collection and genomic analysis was approved by the institutional ethics committee, and all of the subjects provided their written informed consent. No significant sequence variants in *SLC22A12* were found. The sequence analysis of the *SLC2A9* gene revealed a novel homozygous nonsense mutation of g.68G > A in exon 3 (p.Trp23Stop), which resulted in prematurely truncated GLUT9 protein in the patient. No similar loss-of-function mutations have been previously reported. A heterozygous mutation of g.68G > A was also found in the parents and younger brother (Figure 3). The results of the analysis in the patient’s family members suggested an autosomal recessive mode of inheritance (Figure 4).

## Discussion

Hypouricemia is arbitrarily defined as a lower serum uric acid (UA) concentration caused by decreased production or increased excretion, and idiopathic renal hypouricemia (IRHU) is a familial hereditary disease characterized by an increased renal urate clearance caused by an isolated inborn error of membrane transport for urate in the proximal renal tubules. Although most patients with IRHU have no clinical symptoms or complications, the major complications in this disease are urolithiasis and exercise-induced acute kidney injury (EIAKI).

EIAKI associated with IRHU was first reported in 1989 by Erley et al. [10], and a number of cases have been reported to date [8]. However, why renal hypouricemia causes exercise-induced AKI remains unclear. One possibility is that AKI might develop due to acute uric acid nephropathy, and tubular obstruction by uric acid has been suggested as causing AKI [11]. Circulatory disturbance of the kidney is an alternative mechanism, as most renal biopsies in cases of exercise-induced AKI show no uric acid crystallization. Because plasma uric acid is a powerful antioxidant, anaerobic exercise induces an accumulation of oxygen-free radicals, which are vasoconstrictive, and this accumulation can result in a reduced glomerular filtration rate. Uric acid seems to play a protective role in the kidney, and the decreased antioxidant potential in renal hypouricemia might lead to kidney injury caused by ROS [12]. Furthermore, patient with type I xanthinuria [13] presents very low serum uric acid because of deficiency in xanthine dehydrogenase, which catalyzes the oxidation of hypoxanthine to xanthine and also of xanthine to uric acid. Affected individuals pass multiple brownish-yellow stones and surgical extraction would be required in some days. Even though, probands with xanthinuria did not have AKI in their medical history. It suggests [14], that hypouricemia alone, probably, could not contribute to renal injury in patients with primary renal hypouricemia. The powerful antioxidant activity of uric acid might not be the fundamental factor for development of AKI in patients with IRHU.

In the present study, no uric acid crystallization was found in the tubular lumen of the kidney sample obtained 9 and 5 days after the first and secondary onset, respectively, of EIAKI. Thus, the role of a tubular obstruction by uric acid crystallization in EIAKI could be excluded in the present case. Acute tubular necrosis, induced by renal vasoconstriction and/or a reduced glomerular filtration rate (because of volume depletion after exercise and vomiting), might have played a role in the pathogenesis of recurrent EIAKI in the present case, according to the results of both renal biopsies. Figure 3 presents the Scr and serum concentration of uric acid simultaneously during the first and second episodes of EIAKI and at follow-up.

*SLC2A9* was first identified as a novel member of the facilitative glucose transporter family in 2000 by Phay et al. [15]. In 2008, Matsuo et al. [16] identified two loss-of-function heterozygous mutations in *SLC2A9* that caused renal hypouricemia by decreased urate reabsorption on both sides of the proximal renal tubules. Since then, several studies have reported on renal hypouricemia caused by *SLC2A9* mutations (summarized in Table 1). Among these studies, six patients were reported as presenting with EIAKI. Dinour [6] reported that three male Israeli-Arab patients with a homozygous L75R mutation presented with EIAKI. Shima [17] reported one female Japanese patient who presented with EIAKI and PRES (posterior reversible encephalopathy syndrome), caused by compound heterozygous G207X/dupExon1a-11 mutations. Stiburkova [18] also reported two renal hypouricemia patients presenting with EIAKI caused by a homozygous G216R mutation or compound heterozygous G216R/N333S mutations in the *SLC2A9* gene. In the present study, the patient manifested with EIAKI and carried a homozygous g.68G > A mutation; her brother, mother and father did not present with hypouricemia, elevated fractional excretion of uric acid or EIAKI, although all three carried the same but heterozygous mutation. Above all, we conclude that homozygous or compound heterozygous mutations in the *SLC2A9* gene are a prerequisite for presenting with EIAKI in patients with renal hypouricemia. The same phenomenon can also be observed in patients presenting with EIAKI who have *SLC22A12* mutations [19-21]. These results imply that not all mutations necessarily lead to EIAKI and that the possible genotype-phenotype correlation is complex and difficult to determine at the present time. Furthermore, mutational analysis could be a useful indicator for the clinical and prognostic evaluation of patients with renal hypouricemia.

**Table 1:** **Case reports of patients with renal hypouricemia caused by *SLC2A9 *** **mutations**

**Case reports**	**Age (Year)**	**Sex**	**Mutation**	**Ethnic group**	**Serum urate (mg/dL)**	**FEUA (%)**	**Complications**
Matsuo [16] 2008	70	F	p.Arg380Trp*	Japanese	1.5	15.7	No
	43	M	p.Arg380Trp*	Japanese	2.7	14.6	No
	32	F	p.Arg198Cys*	Japanese	2.1	—	No
Anzai [22] 2008	36	F	p.Pro412Arg *	Japanese	2.4	—	No
Dinour [6] 2010	46	M	p.Leu75Arg^#^	Israeli-Arab	0.2	>150	EIAKI
	67	M	p.Leu75Arg^#^	Israeli-Arab	0.67	>150	Nephrolithiasis and CKD
	36	M	p.Leu75Arg^#^	Israeli-Arab	0.04	>150	Nephrolithiasis
	24	M	p.Leu75Arg^#^	Israeli-Arab	0.20	>150	EIAKI
	19	M	p.Leu75Arg^#^	Israeli-Arab	0.10	>150	EIAKI
	10	F	p.Leu75Arg^#^	Israeli-Arab	0.01	>150	No
	69	M	p.delExon7^#^	Ashkenazi-Jewish	0.10	>150	Nephrolithiasis
Stiburkova [7,23] 2011	21	M	p.insCExon3^#^	Czech	0.17	220	No
	16	F	p.insCExon3^#^	Czech	0.17 ± 0.05	194 ± 99	No
Shima [17] 2011	11	F	p.Gly207X/p.dupExon1a-11*^#^	Japanese	0.1	58.3	EIAKI
Dinour [24] 2012	7.5	F	p. Arg171Cys^#^	Israeli-Arab	0.1	138	No
	5.5	M	p. Arg171Cys^#^	Israeli-Arab	0.1	157	No
	2.3	F	p. Arg171Cys^#^	Israeli-Arab	0.2	88.8	No
	84	M	p.Thr125Met^#^	Sephardi-Jewish	0.2	151	No
Stiburkova [18] 2012	12	M	p.Gly216Arg ^#^	Anglo-Saxon	0.20	45.8	EIAKI
	14	M	p.Gly216Arg / p.ASN333Ser*^#^	Anglo-Saxon	0.16	93	EIAKI
Current study 2013	12	F	p.Trp23Stop^#^	Chinese	0.05	295.99	EIAKI
	8	M	p.Trp23Stop/WT*	Chinese	1.94	24.72	No

*Heterozygous, ^#^Homozygous, *^#^Compound heterozygous. FEUA: fractional excretion of uric acid; EIAKI: exercise-induced acute kidney injury; CKD: chronic kidney disease. Reference ranges for uric acid [4]: 2.0 ~ 5.7 for under 15 years and adult female, 2.0 ~ 7.0 for adult male. Reference ranges for FE-UA [4]: 7.3 ± 1.3 for under 15 years and adult female, 10.3 ± 4.2 for adult male. Mean/median value for serum creatinine [25]: 45 μmol/L for 1–3 year, 57 μmol/L for 4–8 year, and 66 μmol/L for 9–17 year.

When a patient suffering from AKI with renal hypouricemia is first seen, diagnosing renal hypouricemia is generally difficult because of the increased level of UA [26] during the acute phase of the disease. In the present study, the serum concentration of uric acid was 201.8 μmol/L during the first episode of EIAKI; thus, we did not realize that the EIAKI was due to renal hypouricemia. After the second episode of EIAKI, the repeated renal biopsy did not supply any convincing evidence, and as we tried our best to identify the cause of the AKI, we finally realized that there might be a positive relationship in this patient between hypouricemia and the two episodes of EIAKI. Therefore, careful attention should be paid to any signs of hypouricemia during the recovery phase of AKI, especially in patients with recurrent AKI. If anyone can realize the correlation between renal hypouricemia and AKI at an appropriate timing, unnecessary renal biopsies must have been avoided in such kind of patients.

Recently, Bhasin et al. reported [27] an 18-year-old white was diagnosed as iRHUC with recurrent AKI after participating in 400-meter race. Before his next race, he was prescribed allopurinol 300 mg daily for 3 days. He completed this race uneventfully. The underlying mechanism for use of allopurinol is to decrease the production of uric acid, then, reducing the filtered uric acid load and lessening the risk of precipitation of the uric acid in the tubules of kidney. Thus, the value of allopurinol to reduce the risk of exercise induced AKI in patients with iRHUC deserves further study.

## Conclusions

Above all, iRHUC is a rare disorder, but it should be considered in patients with EIAKI, especially in those patients who manifest moderately elevated or normal serum concentrations of uric acid during the acute phase of AKI. Mutational screening of the *SLC2A9* gene is necessary for the diagnosis of iRHUC, and homozygous mutations of the *SLC2A9* alleles can cause severe hypouricemia. Careful attention should be paid to any signs of hypouricemia during the recovery phase of AKI and during long-term follow-up.

### Consent

Written informed consent was obtained from the patient’s legal guardian and from the patient for publication of this case report.

For classification according to the rate of decline of glomerular filtration rate, CKD is staged as blow:

Stage 1: GFR ≥ 90 ml/min/1.73 m^2^

Stage 2: GFR 60 ~ 89 ml/min/1.73 m^2^

Stage 3a: GFR 45 ~ 59 ml/min/1.73 m^2^

Stage 3b: GFR 30 ~ 44 ml/min/1.73 m^2^

Stage 4: GFR 15 ~ 29 ml/min/1.73 m^2^

Stage 5: GFR < 15 ml/min/1.73 m^2^ or on dialysis

## Abbreviations

iRHUC: Idiopathic renal hypouricemia; AKI: Acute kidney injury; EIAKI: Exercise-induced acute kidney injury; URAT1: Urate transporter 1; FEUA: Fractional excretion of uric acid; eGFR: Estimated glomerular filtration rate; PRES: Posterior reversible encephalopathy syndrome; CKD: Chronic kidney disease.

## Competing interests

The authors declare that they have no competing interests.

## Authors’ contributions

SHJ, FCY, JX & FHD carried out the molecular genetic studies, participated in the sequence alignment and drafted the manuscript. WX & LAM participated in the clinical diagnosis of the patient. SQ & DLZ participated in the sequence alignment. GWZ & THF participated in renal biopsy and pathology. SHJ & MJH participated for program design, manuscript drafting. All authors read and approved the final manuscript.

## Pre-publication history

The pre-publication history for this paper can be accessed here:

http://www.biomedcentral.com/1471-2431/14/73/prepub
